# A Cyber-Physical System for Near Real-Time Monitoring of At-Home Orthopedic Rehabilitation and Mobile–Based Provider-Patient Communications to Improve Adherence: Development and Formative Evaluation

**DOI:** 10.2196/16605

**Published:** 2020-05-11

**Authors:** Timothy Stevens, Ryan S McGinnis, Blake Hewgill, Rebecca H Choquette, Timothy W Tourville, Jean Harvey, Richard Lachapelle, Bruce D Beynnon, Michael J Toth, Christian Skalka

**Affiliations:** 1 Department of Computer Science University of Vermont Burlington, VT United States; 2 Department of Electrical and Biomedical Engineering University of Vermont Burlington, VT United States; 3 Department of Orthopedics and Rehabilitation University of Vermont Burlington, VT United States; 4 Department of Rehabilitation and Movement Sciences University of Vermont Burlington, VT United States; 5 Department of Nutrition and Food Sciences University of Vermont Burlington, VT United States; 6 Department of Molecular Physiology and Biophysics University of Vermont Burlington, VT United States; 7 Department of Medicine University of Vermont Burlington, VT United States

**Keywords:** device use tracking, internet of things, neuromuscular electrical stimulation, exercise, smart devices, mHealth, rehabilitation, mobile health, digital health

## Abstract

**Background:**

Knee extensor muscle performance is reduced after lower extremity trauma and orthopedic surgical interventions. At-home use of neuromuscular electrical stimulation (NMES) may improve functional recovery, but adherence to at-home interventions is low. Greater benefits from NMES may be realized with closer monitoring of adherence to at-home prescriptions and more frequent patient-provider interactions.

**Objective:**

This study aimed to develop a cyber-physical system to monitor at-home adherence to NMES prescription and facilitate patient-provider communications to improve adherence in near real time.

**Methods:**

The RehabTracker cyber-physical system was developed to accomplish this goal and comprises four components: (1) hardware modifications to a commercially available NMES therapy device to monitor device use and provide Bluetooth functionality; (2) an iPhone Operating System–based mobile health (mHealth) app that enables patient-provider communications in near real time; (3) a clinician portal to allow oversight of patient adherence with device use; and (4) a back-end server to store data, enable adherence analysis, and send automated push notifications to the patient. These four elements were designed to be fully compliant with the Health Insurance Portability and Accountability Act. The system underwent formative testing in a cohort of patients following anterior cruciate ligament rupture (n=7) to begin to assess face validity.

**Results:**

Compared with the NMES device software–tracked device use, the RehabTracker system recorded 83% (40/48) of the rehabilitation sessions, with 100% (32/32) of all sessions logged by the system in 4 out of 7 patients. In patients for whom tracking of automated push notifications was enabled, 100% (29/29) of the push notifications sent by the back-end server were received by the patient. Process, hardware, and software issues contributing to these inaccuracies are detailed.

**Conclusions:**

RehabTracker represents a promising mHealth app for tracking and improving adherence with at-home NMES rehabilitation programs and warrants further refinement and testing.

## Introduction

### Background

Traumatic injury to the knee joint, including rupture of the anterior cruciate ligament (ACL), is common and highly debilitating [1]. Despite surgical reconstruction and rehabilitation, many patients suffer muscle weakness following the index trauma and surgical intervention that persists for years after surgery [2,3] and are not satisfied with their knee functionality [4]. Current rehabilitation regimens are designed to restore muscle function to preinjury or presurgery levels but are only marginally effective [5]. This may be due, in part, to how pain, impaired neural activation, restricted range of knee motion, and risk for damaging the healing ACL graft limit the rehabilitation regimens available in the early, postinjury, and postsurgical periods. There is a need for improved rehabilitation modalities that can be used at these times to mitigate atrophy and weakness and improve long-term function.

Neuromuscular electrical stimulation (NMES) is an ideal candidate intervention for the early postinjury and postsurgical periods. This patient-directed therapy uses a portable, hand-held device to initiate muscle contraction by passing current through electrodes placed over the muscle of interest. NMES is effective at preventing skeletal muscle atrophy caused by experimentally induced muscle disuse in closely monitored research studies [6,7] and is approved by the Food and Drug Administration (FDA) for this indication following injury and surgery. In fact, studies show that it prevents quadriceps weakness and atrophy following ACL rupture and surgical reconstruction [8,9] and enhances long-term functional recovery [10]. However, use of NMES in orthopedic patients in the postinjury and early postsurgical periods may be limited by the need for associated costly outpatient clinic visits. Although NMES devices are amendable to home use, low adherence to home-based rehabilitation interventions [11-13] have tempered enthusiasm for its use in this setting. Some NMES devices allow for covert monitoring of adherence in the device software that provides the capacity to track at-home NMES use; however, as this oversight is retrospective, there is no opportunity to intervene and correct nonadherence as it occurs. New tools for administering and monitoring rehabilitation may improve adherence with at-home interventions such as NMES by allowing closer provider oversight and facilitating patient-provider interactions to address specific adherence issues.

Sensors and communication equipment can be coupled to medical devices to form cyber-physical systems that allow for near real-time monitoring of treatment adherence and clinical status as well as provide novel opportunities for patient-provider communication. As of 2018, over 95% of adults in the United States owned a cell phone [14], suggesting tremendous potential for cyber-physical systems to improve treatment adherence and monitor health outcomes. Such systems have been developed to monitor glucose levels in diabetics [15] and posture and joint loading in patients following hip surgery [16], to name a few. In patients recovering from musculoskeletal injury and surgery, efforts to develop cyber-physical systems to aid rehabilitation have been limited. Recent reports describe Web-based resources to assist patients with self-guided, at-home orthopedic rehabilitation [17]. To our knowledge, however, no reports have described the construction of a cyber-physical system to support adherence monitoring of at-home rehabilitation with NMES.

### Objectives

To address this technological gap as well as the clinical needs of patients and rehabilitation professionals, we sought to develop a cyber-physical system, comprising an instrumented medical device, a mobile health (mHealth) app, and back-end server architecture, to monitor and improve adherence with at-home NMES therapy. Along with the basic functionality, a crucial feature of our system is compliance with the Health Insurance Portability and Accountability Act (HIPAA), which is a law that requires strict protections for the storage of and access to protected health information. In addition, we performed initial formative testing of the system in patients following ACL injury to assess its functionality in a real-world setting and discern whether the system would be suitable for further development and testing.

## Methods

### System Components

**Figure 1:**
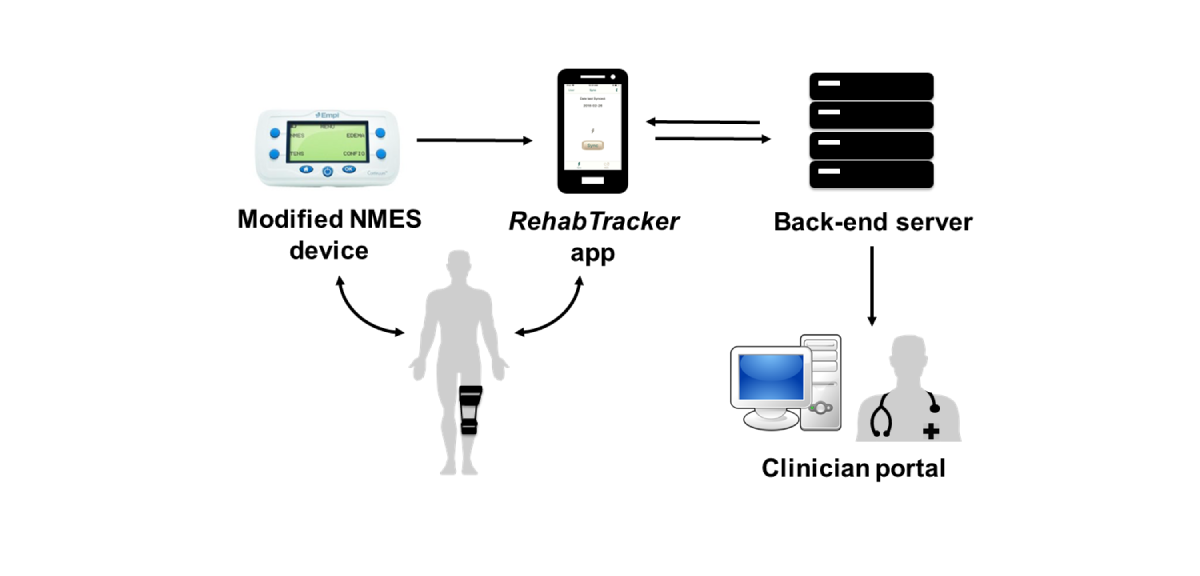
Simplified overview of the components of the RehabTracker cyber-physical system and user interactions. The RehabTracker mobile health (mHealth) app receives and transmits neuromuscular electrical stimulation (NMES) use data and serves as a conduit for patient-provider interactions. A secure, Health Insurance Portability and Accountability Act–compliant, back-end server receives the device use data, displays the adherence data for care provider review, and sends automated push notifications to the mHealth app, with the goal of improving adherence to the NMES prescription. NMES: neuromuscular electrical stimulation.

The RehabTracker cyber-physical system comprises four main components (Figure 1): (1) the modified NMES device, (2) the iPhone Operating System (iOS) mobile app (mHealth app), (3) the back-end server, and (4) the clinician portal Web interface. As patients perform NMES with the modified device, rehabilitation session data are recorded. After each session, patients use the RehabTracker app to transfer the session data from the embedded device to the database. In turn, the Web-based data are viewed by the clinician and used for automated adherence tracking and push notifications. The following sections detail each component of the system.

### Neuromuscular Electrical Stimulation Hardware and Software Development

**Figure 2:**
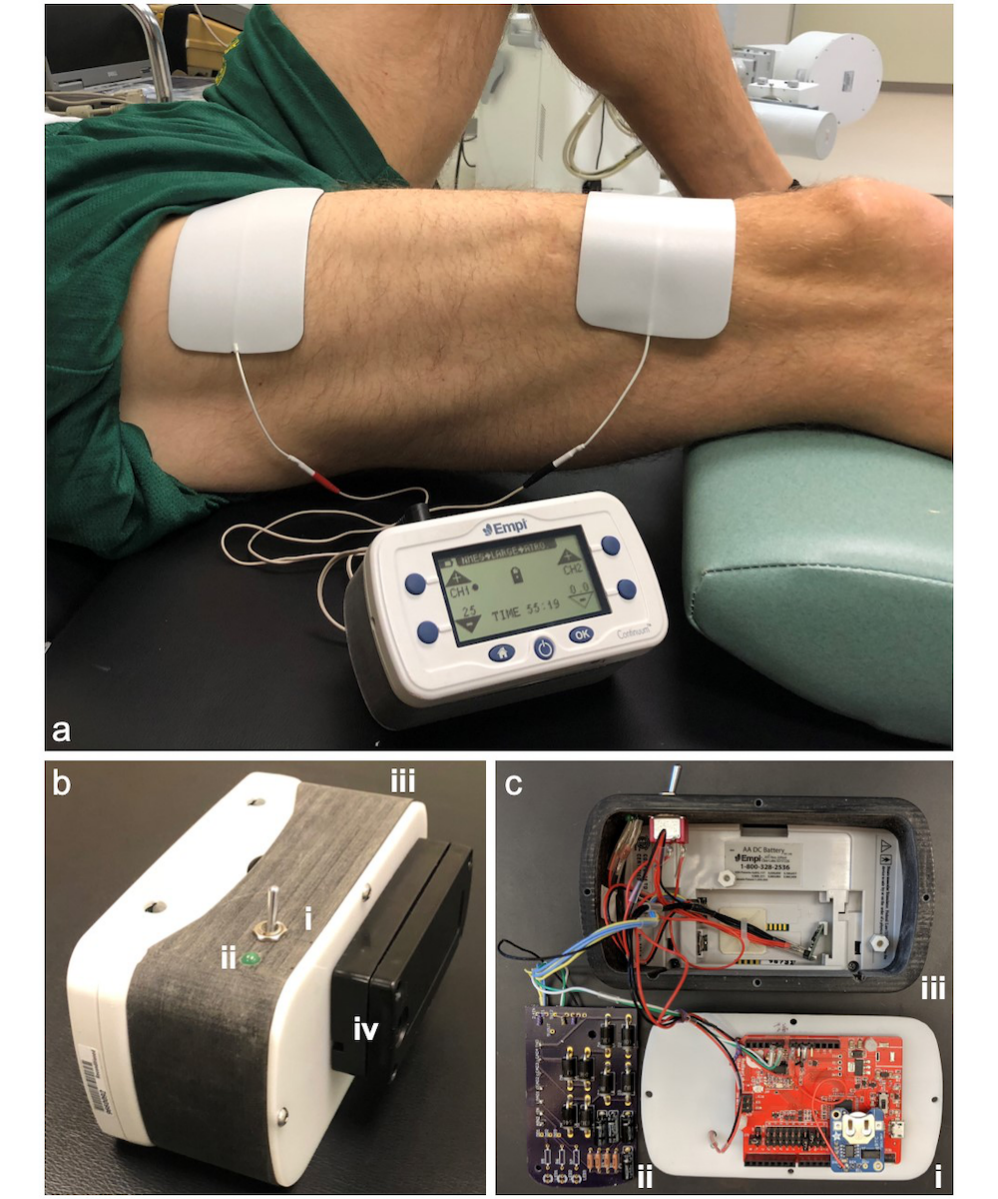
Modified neuromuscular electrical stimulation system can track the duration, intensity, and timing of rehabilitation sessions (labeled as “a”). Exploded view of the system, comprising a RedBearLab’s Blend BLE (labeled as “c-i”) and custom circuitry for quantifying device usage (labeled as “c-ii”) and integrated within a 3D-printed enclosure secured to the back of the EMPI (labeled as “b-iii”). Power is provided by two AA batteries secured in an external housing (labeled as “b-iv”) and is controlled by a master switch (labeled as “b-i”) and step-up regulator (labeled as “c-iii”). An external light-emitting diode (labeled as “b-ii”) indicates when the device is powered on.

The core of this system is the Empi Continuum (Figure 2), an FDA-cleared multifunctional electrotherapy device that offers adjunctive electrophysical rehabilitation therapies, including the NMES therapy considered herein. The main goals of the modifications to this system were (1) to render it capable of tracking device use and (2) communicate data to a companion iOS app via Bluetooth 4.0.

The instrumentation to achieve both these goals is built upon the RedBearLab’s Blend BLE (Figure 2), a small development board that includes an integrated microcontroller and Bluetooth 4.0 module. The four output leads of the EMPI device are passed through a custom rectifier and voltage divider circuit (Figure 2) and sampled through two analog inputs of the Blend. The resulting signal provides a direct measure of device activity that is logged quantitatively using custom firmware on the device. The Blend was also integrated with a real-time clock to provide absolute time stamps that were used to characterize the duration of each rehabilitation session. All this information is communicated to a mobile phone via Bluetooth low energy.

The combined Blend-EMPI system is powered by two AA rechargeable nickel metal hydride batteries and is controlled by a master switch (Figure 2), ensuring that a rehabilitation session cannot be completed without also being tracked by the monitoring hardware. A step-up regulator (Figure 2) is used to provide the requisite 5 V to the Blend. The EMPI’s internal low-voltage cutoff is retained, ensuring that the system cannot be used if the batteries are no longer capable of providing sufficient power to enable a standardized rehabilitation session. If this state is reached, an indicator light-emitting diode (Figure 2) will not illuminate when the master power switch is engaged. The Blend system is enclosed within a 3D-printed housing that is secured to the back of the EMPI (Figure 2). The batteries are housed in an external enclosure that allows for easy replacement by the user and acts as a *kickstand* for the device, when in use (Figure 2).

The Blend firmware serves two purposes: (1) processes the voltages from the NMES device into session data and (2) sends the data to the RehabTracker mHealth app via Bluetooth. During a therapy session, the NMES device outputs alternating waves of high and low voltage, which trigger muscle contraction and relaxation, respectively. When enabled, the Blend constantly monitors the NMES device voltage and automatically identifies the beginning and the end of a therapy session as recorded by an onboard real-time clock based on a simple threshold-based state machine. As a measure of the session intensity, an average maximum voltage variable is calculated by determining the peak voltage achieved during each muscle contraction cycle and averaging across the session. When a session is completed, the data for that session (start time, end time, and average peak voltage) are stored in the Blend’s local storage until a sync is initiated by the user. Data storage space is not an issue as the Blend’s 256 KB local storage can hold the data of far more sessions (approximately 24 bytes per session) than a patient would complete. Once a sync is initiated by the user, stored session data are sent to the RehabTracker mHealth app and deleted from the Blend’s storage. Data transmission from the modified NMES device to the mobile app is enabled by the Blend firmware and is transferred in a custom format.

### Mobile Health App Development

**Figure 3:**
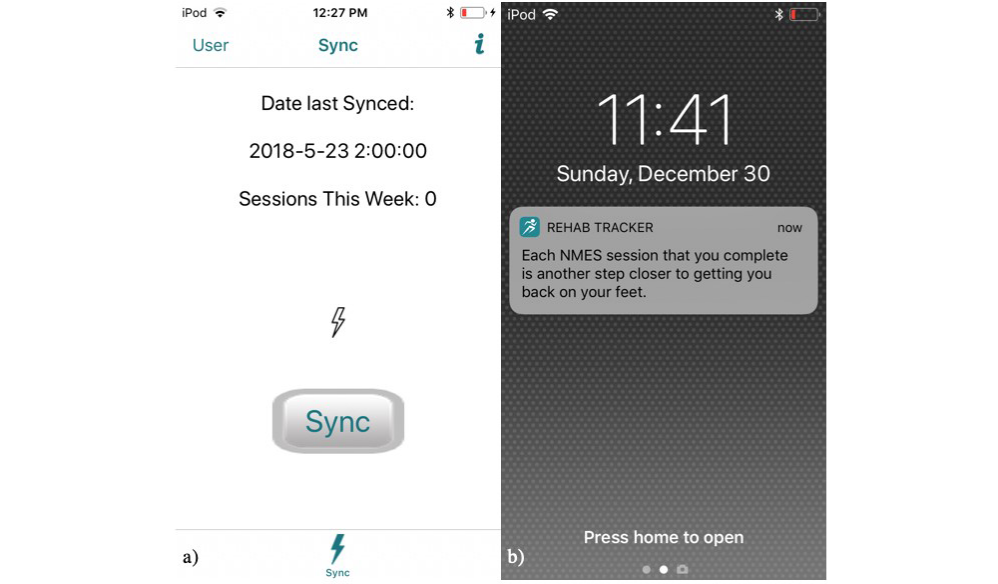
Sample screenshots from the RehabTracker mobile health app, which includes functionality to sync session data with the secure database (shown in screenshot “a”) and encourages patients’ adherence to the push notifications (shown in screenshot “b”).

Patients predominantly interact with the system through the mHealth app. The app allows patients to view and sync their session data (Figure 3) to the database. Communication with the patient via push notifications is also supported by the app (Figure 3). The app was developed for iOS because of its high preference in the age demographic most likely to sustain traumatic knee injuries [18]. However, all supported functionality could be replicated on the Android platform without technical barriers.

#### App Interface

The most significant feature of the mobile app is the sync feature (Figure 3), which sends patient data from the modified NMES device, through the app, to the database via our representational state transfer (REST) application programming interface (API). When a patient presses the sync button on the app, it initiates the Bluetooth scanning procedure in an attempt to establish a connection with any nearby modified NMES device. The process occurs from within the app, and patients do not need to pair their phone with their NMES device. After establishing a connection, the app reads session data from the device’s Bluetooth characteristic until it reaches the end-of-message symbol or the connection times out after 30 seconds. Parenthetically, we did not include a layer of security that required the modified NMES device to be paired with a specific user mobile device as the close oversight of the software and device disbursement in this study meant that the likelihood of the two devices and/or apps being in close proximity was extremely remote. Nonetheless, future versions will incorporate device pairing.

Data collected from the NMES device are parsed into JavaScript Object Notation objects and stored locally on the patient’s phone. The app also attempts to upload these locally stored sessions to the database via our REST Web API. On-phone data storage is only used to prevent data loss from network errors or when internet service is unavailable. These copies are deleted after the data are successfully uploaded to the database. In addition to the one-button sync feature, the iOS app also provides user authentication for patients. This introduces additional security to the app and ensures that the data synced through the app will only be associated with the logged-in user.

#### Push Notifications

**Figure 4:**
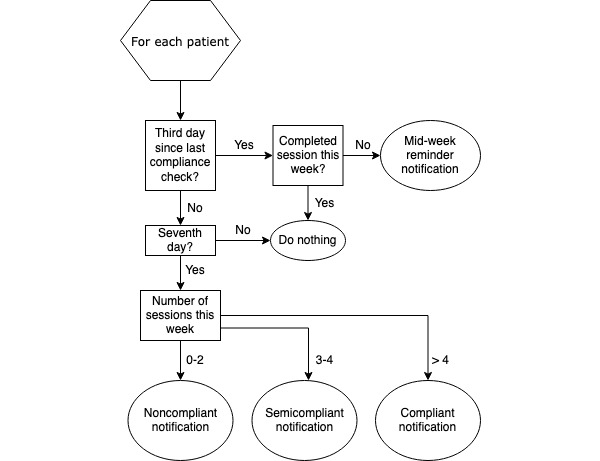
Patient adherence evaluation algorithm. Adherence is checked twice weekly. Positive and remedial push notifications are sent based on the number of neuromuscular electrical stimulation sessions completed.

RehabTracker uses automated push notifications to communicate positive and motivational messages to patients on an almost daily basis, an approach designed to reduce overhead time for clinicians in the daily process of communicating with patients regarding adherence to their rehabilitation prescription. The notification method, based on social cognitive theory [19] and outlined in Figure 4, reminds and encourages patients to complete NMES sessions, without contacting them so frequently that they ignore notifications. There are three events that initiate a push notification, including (1) a completed session, (2) a week starting with no sessions, and (3) the end of a week. For each type of event, a message, randomly chosen from an extensive set of messages associated with that event and the patient’s level of adherence, is sent to the patient via an app notification. Mixing up the messages and assuring that there is sufficient number of messages make the experience less automated. The examples of message content for each event type are listed in Textbox 1, and a screenshot of how the notifications appear to patients is presented in Figure 3. Generally, the messages are designed to enhance motivation and encourage the patients’ confidence in their ability to comply with rehabilitation.

**Examples of text used in different types of notifications sent to patients.**

After session

- “Great job finishing your NMES session!”

Compliant

- “Fantastic week! You met your goal for NMES sessions. Keep up the good work!”

Semicompliant

- “We would like to see you complete 5 NMES sessions per week. You did great last week but didn’t quite sync 5 sessions. Is there anything we can do to help?”

Noncompliant

- “Tough week? We saw that you did not sync 5 sessions of NMES. Let us know if there is anything we can do to help.”

Midweek reminder

- “We noticed you have not completed an NMES session in the last 3 days. Is there a problem? Can we help? Give us a call”

After a patient syncs session data with the system, they receive a positive reinforcement notification from the system. As patients should be completing five sessions every week (each 7-day period starting with the first day of RehabTracker system use), these are the most frequent notifications. If a patient begins their week with three consecutive days without syncing a session, then a reminder notification is sent inquiring if they forgot to sync their sessions or if they are having problems with the device or app. Finally, at the end of a patient’s week, the patient receives a notification reporting their total adherence for the week. We have created three subcategories for patient adherence notifications for these weekly communications: (1) compliant (>4 sessions completed), (2) almost compliant (3-4 sessions completed), and (3) noncompliant (<3 sessions completed). This classification scheme ensures that the patients who are completing some, but not all, of the required sessions receive positive reinforcement while also being nudged to improve adherence during the next week. In noncompliant patients, care providers are alerted to the patients’ noncompliance so that additional patient-provider communication can be undertaken to resolve issues contributing to the nonadherence.

### Clinician Portal Web Interface Development

**Figure 5:**
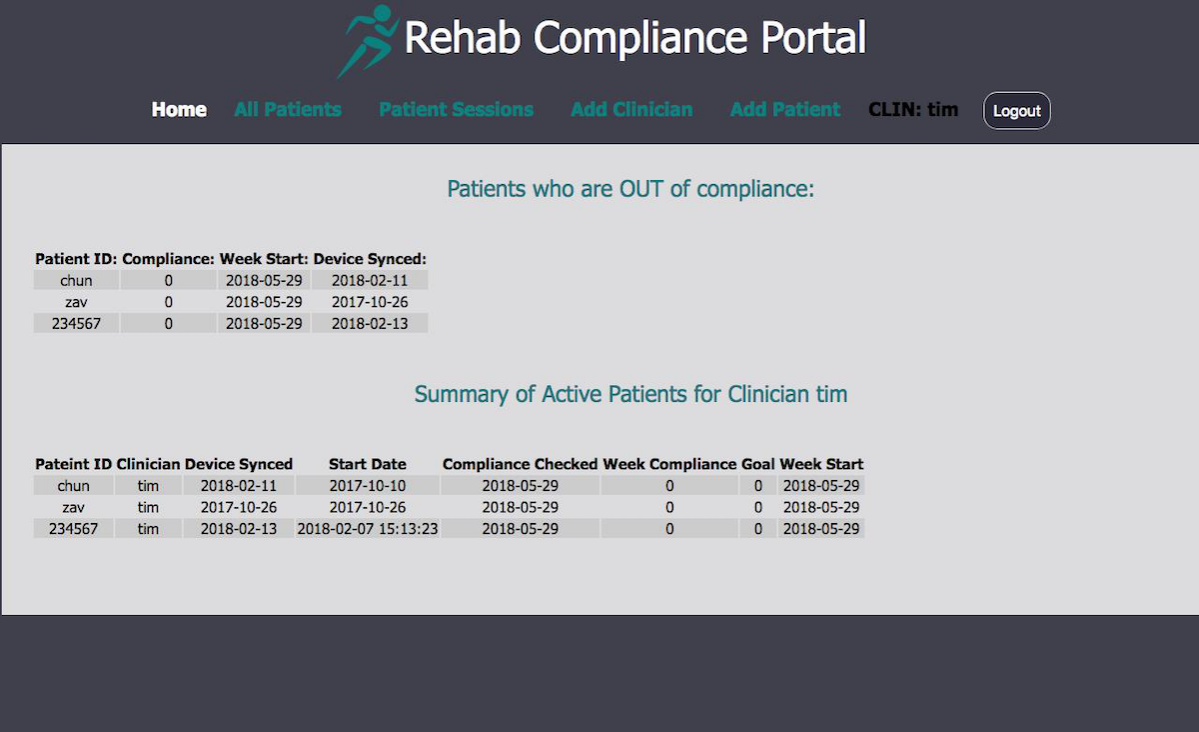
Sample screenshot of the clinician portal, which identifies patients who are nonadherent; provides summary information on the current patients; and offers functionality to view all patients, individual patient sessions, and to add clinicians (administrator only) and patients.

The clinician portal provides near real-time access to patient adherence and session data. The site’s front end is written in hypertext preprocessor (PHP) language and directly interfaces with the MySQL database. Queries are sanitized by our structured query language (SQL) database API. The site is also hosted with hypertext transfer protocol secure (HTTPS) to ensure HIPAA compliance. Data are immediately updated after a patient’s data sync. Upon logging in with a username and password, clinician users receive various views of their patients’ data (Figure 5). The home screen sorts patients by their adherence status to alert clinicians of noncompliant patients who may need more attention. Clinicians can also see device use data on a session-specific level. The portal also allows clinicians to enroll patients into RehabTracker and allows admin clinicians to add other clinicians.

### Back-End Server

The back-end server that enables the RehabTracker cyber-physical system includes the following components/features: (1) REST API, (2) MySQL database, (3) adherence assessment, (4) push notifications, and (5) HIPAA adherence and security. Each component is described in more detail below.

#### Representational State Transfer Application Programming Interface

The RehabTracker iOS mobile app follows a 3-tier client-server architecture when interacting with the database. For the middle tier, we implemented a REST API in PHP. The primary functions of the RehabTracker cyber-physical system are enabled by this API. When a patient presses the sync button in the iOS app, all session data are transferred through the API to the database. It also creates a push notification for the patients when they sync, to provide immediate positive feedback. Additional features of the Web API include authentication of users.

#### MySQL Database

A MySQL database was used to store data on a HIPAA-compliant server. A total of five tables store and relate clinician, patient, session, push, and notification data. The patient and clinician tables store information about patients that will remain constant throughout the study. When a patient completes a session, a new row is added to the session table to log it, and whenever a new push notification is sent, it is added to the push table. The notification table contains the text of the push notification ordered by the push notification category.

#### Adherence Assessment

RehabTracker automatically checks a patient’s adherence on the third and the seventh day of each week relative to their start date (Figure 4). A scheduled server process performs these checks. According to the NMES prescription for these patients, adherence is defined as the completion of a 1-hour session each day for 5 days every week. The push notification regime associated with this adherence checking is discussed in detail above.

#### Push Notifications Infrastructures

The RehabTracker server uses three components to generate, store, and send Apple push notifications (APNs). The adherence script and API generate push notifications based on the user data, the database stores the notifications, and a scheduled server job sends the notifications with APN. The data sync API endpoint and adherence script described above upload push notifications to the database. Each notification is stored in the database with the ID of the patient who is receiving the notification and the index of the notification body.

Every hour, the push notification script queries the database for all unsent notifications, builds notification objects with the notification bodies and the universally unique identifier of the patients’ phones, and sends these notification objects to users with APN. Notifications are stored in the database agnostic of purpose; that is, with regards to adherence or completed session. Accordingly, the same notification-sending script is used for both. The push notification script is written in Python and takes advantage of the Python APNs library for sending APNs. The script also uses the REST API to receive all information from the database. This minimizes exposed database credentials and ensures that the messages are encrypted when sent from the database to the script.

#### Health Insurance Portability and Accountability Act Compliance and Security

All patient data in RehabTracker are anonymous. When a patient is signed up to use the RehabTracker, they receive an anonymous user ID from the clinician with no correlation to their personal information. The patient uses this ID as their RehabTracker username, and all data in the database for that patient is related to this ID. This approach ensures that data stored in the database are anonymous, and, thus, are not protected health information, as defined by HIPAA [20]. The app itself does not use passwords. Instead, users log in with their anonymous ID. In addition, the use of the app requires the customized NMES device. All these elements further reinforce data security.

The database, Web API, and provider portal are hosted on a HIPAA-compliant server. The database uses Research Electronic Data Capture to secure permanent data storage. To access the data, one must use the Web API or provider portal. Each of these points of access actively limits the SQL injection to minimize the data one may access. The API and portal are also implemented using HTTPS, taking advantage of the transport layer security encryption. In addition, the provider portal is username and password protected. Only developers have root privileges. These security and privacy measures have allowed us to test RehabTracker in the clinical setting.

### Pilot Clinical Study

We undertook a formative evaluation of the RehabTracker system on a convenience sample of patients who were taking part in a clinical trial of NMES use in patients who had suffered ACL injury and undergone surgical reconstruction (NCT02945553).

#### Participants

A total of 7 patients (3 women and 4 men) were selected to use RehabTracker. All patients were aged between 18 and 50 years; had BMI <35 kg/m^2^; had suffered an acute, unilateral, first-time ACL rupture; and were scheduled to undergo reconstructive surgery. Patients were excluded based on the following criteria: (1) history of knee injury/surgery of either leg or nonsurgical intervention; (2) abnormal laxity in any other lower extremity besides the injured ACL; (3) signs or symptoms of arthritis, autoimmune or inflammatory disease, or diabetes; (4) grade IIIb or greater articular cartilage lesions; and (5) women who were pregnant or planning on becoming pregnant. Written informed consent was obtained from all volunteers before their participation, and all protocols and procedures were approved by the Committee on Human Research in the Medical Sciences at the University of Vermont.

#### Procedures

Patients (age: mean 22 years, SE 1; BMI: mean 26 kg/m^2^, SE 1) used RehabTracker for 1 to 2 weeks, performing between 5 and 10 NMES rehabilitation sessions. The RehabTracker system was used after a patient had experience using NMES therapy so that the errors in use of the device would not affect the usability of the RehabTracker system. Each patient was enrolled in the RehabTracker study by a single participating clinician. Following enrollment, the RehabTracker app was downloaded to their personal iOS device using Apple’s TestFlight service. The download links were sent to an email address created as a part of the enrollment procedure. While using the RehabTracker system, patients simultaneously logged their NMES use on paper-based log sheets. Patients’ self-report logs were kept to document the NMES device use and were compared with the use data recorded by RehabTracker to verify the functional correctness of the system for recording NMES sessions.

## Results

### Functional Correctness

We conducted an initial assessment of the functional correctness of the system, specifically, its ability to perform the two main functions for which it was designed: (1) to sense and convey information about the NMES device use to the back-end server/clinician portal and (2) to function as a platform for provider-patient communications about device use adherence. Numerical results are provided as these are the basis for the assessment of functionality; however, we acknowledge that these numbers reflect the prototype functionality rather than the true adherence to the NMES prescription. Nonetheless, our efforts to define functionality uncovered issues that will be addressed in future prototypes.

With regard to the first design goal of our system, before we could define the aspects of the system that functioned properly and the ones that did not, we had to define whether the sessions that the patients completed were tracked by our system. Of the total number of sessions that the patients self-reported using the modified NMES device, 75% (55/73) were recorded by the RehabTracker system. In 2 patients, 100% (26/26) of the self-reported sessions were tracked by the system, whereas 2 patients had ~90% (20/22), one patient had 75% (6/8) and one patient had 23% (3/13) of the self-reported sessions tracked by the system. Finally, in another patient, none of the sessions (n=4) were recorded as the modified NMES device was not synced with the RehabTracker app. This failure is likely related to the patient not using the device, as no sessions were recorded by the device use tracking feature of the NMES device stock software. Thus, excluding this last patient, 80% of the patient-reported sessions were recorded by the system.

We initially chose patient-reported NMES device use as our comparator for RehabTracker system functionality. However, in the home environment, patient-reported adherence data may be less reliable [11]. To explore this possibility, we used the covert monitoring feature built into the EMPI Continuum software that tracks device use. Using this device use monitoring feature, results from our broader trial with nonmodified EMPI Continuum devices showed that patients overreported device use by approximately 12%. Overreporting of device use by patients could cause bias in our assessment of the functionality of the RehabTracker system, specifically toward the RehabTracker recording less sessions compared with patient-reported device use.

Owing to this potential bias in self-report, we also examined the ability of the RehabTracker system to monitor home NMES device use by comparing the sessions recorded by the system with those logged by the device use monitoring feature of the software. This approach uncovered an issue with our device modifications on the NMES device’s internal software. In two patients, the number of device-reported sessions were spuriously high (34 and 135 sessions), suggesting that, in some cases, the NMES device modifications made for the RehabTracker system interfered with the covert monitoring feature such that it did not accurately record the number of sessions completed. Not considering these two patients, the device software reported 48 total sessions completed by the remaining patients. For these remaining patients, the self-report records showed 55 sessions completed, which represents a 15% (55/48) overestimate. This estimate is in accord with the data from the unmodified devices used by other participants throughout the remainder of the trial showing overreporting bias. The RehabTracker system reported 40 sessions for these same patients, which corresponds to 83% (40/48) of the device-reported sessions.

With regard to the second goal of providing a platform for communications aimed at improving device use adherence, patients for whom the tracking of automated push notifications was enabled (n=3), received 100% (29/29) of their expected push notifications. An additional 2 patients reported receiving push notifications; however, we did not have records that they received these notifications as the patients’ device ID became dissociated from our system log because of log-ins from different devices. In addition, two patients did not receive the push notifications as the notification-sending script was not scheduled to run during their system participation. The system functioned properly for patients with complete notification logs.

### Other Software and Process Issues

We discovered software issues with the mHealth app during formative testing. In the first version, a bug in the authentication logic prevented users from logging in. This was fixed, but it delayed the patients’ use of the system. In addition, the patient enrollment and app installation procedure required coordination among the developers and study coordinators and several distributed steps. The added complexity of this process delayed the patients’ participation.

### Hardware Issues

Several hardware issues were uncovered with testing. First, the peak NMES device voltage logged by the RehabTracker did not match with the patients’ self-reports of intensity. One volunteer did not self-report the device intensity and another did not sync sessions, leaving 5 volunteers for comparison. Among these patients, the RehabTracker’s intensity matched the patient-reported intensity on average (36, SD 13 vs 36, SD 2 arbitrary units, respectively), but did not match on an individual basis (*r*=−0.20; *P*=.75). This individual inaccuracy is likely related to the high degree of variability in the device intensity recorded by the RehabTracker device. Second, the modifications to the stock NMES device to enable the RehabTracker system to monitor device use created problems with the NMES use tracking feature available as part of the stock device software. As reported above, in two patients, the number of device use sessions were spuriously high (34 and 135). The reason for this error was not readily apparent and will require further testing. Third, one patient stopped using the modified NMES device when the batteries ran out as the patient felt that the battery case was too cumbersome to open. Finally, there was a problem with the real-time clock that caused clock drift. We addressed this by time-stamping data in the app as they were received from the device, using time on the device hosting the app instead.

## Discussion

### Principal Findings

Knee extensor muscle performance is profoundly reduced in the postinjury and early postsurgical period following ACL reconstruction [3,21] because of a combination of neural, biomechanical, and pain limitations. Although orthopedic rehabilitation aims to prevent or remediate these maladaptations, most fall short of this goal, as evidenced by persistent atrophy and weakness in the years following surgery [3,22,23]. This loss of strength could have consequences for the development of future joint pathology [3], and interventions to prevent its development may contribute to better health outcomes and restoration of normal lower extremity function.

One potential reason why many rehabilitation programs do not remediate the loss of strength and function to preinjury levels is that a large proportion of the prescribed therapeutic exercises and activities are performed at home, particularly during the early postsurgical period. In this environment, there is little or no oversight by the rehabilitation practitioners. Greater functional improvements may be realized with closer oversight by clinicians and more frequent provider-patient communications. In particular, approaches that facilitate patient-provider communications and seek to improve adherence could prevent strength deficits from developing. To this end, we described the construction of a cyber-physical system that enables the monitoring of home-based NMES use and an mHealth app that facilitates communication of device use data to clinicians and provides a platform for automated positive and remedial messages via push notifications to patients who are geared to improve device use adherence.

This study builds on our prior work to develop early rehabilitation programs for patients suffering ACL injury who have undergone surgical reconstruction [24,25]. In the prior work, rehabilitation was performed during clinic visits and was supervised by study personnel. Although this is a rigorous approach to test the safety and efficacy of such interventions, it is impractical in a real-world clinical setting. NMES has shown promise in preserving muscle size and function [8,9] and enhancing long-term functional recovery [10] in orthopedic surgical patients. The use of NMES over extended periods of time after injury and surgery may be problematic, however, as much of this rehabilitation would need to be performed at home, where adherence is generally low. Moreover, adherence with rehabilitation interventions decreases over time [25]. The cyber-physical system described here seeks to address these issues and improve adherence.

A goal of the RehabTracker system is to enhance the patient-provider communication to improve adherence with NMES device use prescription. Accordingly, our approach for these communications deserves some discussion. The patients’ psychological responses to knee injury, surgery, and/or rehabilitation may be important for their ability to return to prior levels of activity and function [26] and, in turn, their satisfaction with surgical outcomes. Self-efficacy, defined as an individual’s perceived ability to successfully engage in targeted behaviors, is associated with rehabilitation adherence and functional outcomes in patients who experience ACL trauma and undergo surgical reconstruction [27]. With this in mind, the design of our communication system was grounded in social cognitive theory [19], which emphasizes improving patients’ self-efficacy toward adherence with rehabilitation prescriptions. Evidence shows that this approach supports adherence to the targeted behaviors for weight loss [28], diabetes management adherence [29], cancer screening [30], and smoking cessation programs [31].

We used a hybrid communications approach that included both automated messaging as well as weekly provider phone calls to patients. The admixture of manual and automated communications could be modified according to the number of outpatient rehabilitation visits, patient needs, and/or insurance coverage for telehealth services. The automated messaging is the most frequent contact and is designed to alleviate burdens on clinicians to monitor patient adherence on a daily basis, while also providing early positive feedback to patients for their adherence to the NMES use prescription. Short messaging service, or texting, has been used more widely than push notifications in health care, including preventative care [32], disease management [33,34], and patient education [35] applications. However, the effectiveness of these interventions has been difficult to judge because of the poor quality of the evidence [36,37]. Although they have been less studied, we used push notifications as they allow more control over the appearance of notifications and support integrating functionality into the notifications (eg, to launch the app). Push notifications also do not require the patients’ cell phone numbers, reducing the possibility of identifying patients using the data in the database, which raises privacy concerns that may influence the patients’ attitude toward using the app.

One goal of this study was to conduct an initial assessment of the functionality of the system. With regard to the goal of tracking at-home NMES use, RehabTracker functioned as designed for most of the patients. Most patients were able to successfully use the RehabTracker cyber-physical system to sync data to the database and received push notifications from the automated communication system. In this context, our initial prototype of the system shows promise and warrants further development and testing. However, we noted process-oriented, hardware, and software shortcomings that are areas for improvement.

The two major system usage problems were largely because of process-oriented issues. In the first instance, problems were encountered by patients setting up the app. A total of 5 patients set up RehabTracker in a knowledgeable clinician’s presence. All of these patients were able to use the RehabTracker device for the duration of their trial period without problem. The first two patients who set up RehabTracker independently had difficulties. We initially allowed patients to enroll independently through the Apple beta testing program, TestFlight, to simulate real-world usage. However, this process was more involved than downloading a traditional iOS app and proved too difficult for these patients to perform independently. Following these difficulties, one patient did not feel confident that the sync procedure downloaded data from the NMES device to the app and the back-end server, which affected their use of the system. In the second issue, when one patient’s modified NMES device ran out of batteries, they switched to a backup device rather than replacing the batteries. Both incidents represent usability concerns for the system, albeit minor ones that can be corrected with hardware (ie, easier access to battery compartment) and software (ie, patient setting up the app with clinician/study personnel and better user notification to confirm data sync) adjustments.

Our ability to identify the functionality of software, firmware, and hardware components of the RehabTracker system was influenced by the fact that we used a convenience sample from an on-going clinical trial to assess the functionality. Imprecision in tracking at-home use of the NMES device hampered assigning specific functionality defects as user- or system-specific. Despite this problem, our study identified several issues with the initial prototype of this system that provide valuable information for future modification.

As data from our broader trial showed that patients overreport NMES use, we used the device use tracking feature built into the NMES device software for comparison with the device use recorded by our system. However, in two patients, we discovered that this was affected by the hardware modifications made to the device to enable the RehabTracker system to track device use. The nature of this interference is unclear and will necessitate further testing and likely redesign of the sensor system for tracking NMES current outflow. More broadly, this initial functionality assessment reveals that our study, similar to many in the field [11], lacks an accurate and objective criterion for assessing at-home exercise/rehabilitation participation and, in turn, functionality and usability of mHealth systems designed to track at-home interventions. Accordingly, future efforts to evaluate the system’s functional correctness will need to incorporate initial laboratory-based assessments to test the usability and functionality of the hardware, firmware, and software before assessments in the home environment.

Our sensor system also did not accurately track the NMES device’s current delivery on an individual basis. Parity in these measures was not a primary design goal as the NMES device intensity varies substantially from day-to-day following injury and in the early postsurgical period, with changes in the level of fluid infiltration in the surrounding tissue (ie, edema). Nonetheless, some measurable NMES device intensity serves as a verification of the device use, and the sensor system can be further refined to improve accuracy in future iterations. In addition, we noted problems with the real-time clock that were likely because of a design flaw in the battery connection of the real-time clock utilized in our prototype. Both of these issues can be addressed in the next prototype iteration by using higher fidelity components to improve upon the accuracy and precision of recording device use.

With regard to the goal of the system to communicate with patients via push notifications, our system functioned largely as designed. That is, sessions that were successfully monitored by the system initiated the automated push notification communications protocol designed to improve adherence to the NMES device use. We did, however, note several process-oriented issues that prevented us from documenting and confirming the receipt of push notifications, which will be fixed in future testing.

### Conclusions

The cyber-physical system that we describe provides a system to collect data on home-based NMES use and communicates NMES device use data from patients to the clinical environment in a HIPAA-compliant, noninvasive manner in near real time. These collected data can be reviewed directly by care providers in the clinician portal and are also available to automated subsystems for actively tracking and improving patients’ adherence through positive and remedial push notifications. Our system differs from prior work in this field that focused on telehealth systems to aid in the performance of classical home rehabilitation [17,27,38]. In contrast, RehabTracker seeks to provide an mHealth-assisted adjunctive rehabilitation modality for patients recovering from orthopedic surgery to supplement existing at-home programs along with an automated messaging intervention grounded in social cognitive theory and designed to improve adherence. With further advancements and testing, the RehabTracker system has the potential to improve provider monitoring of patients’ adherence with at-home NMES prescription.

## Abbreviations

ACL: anterior cruciate ligament
API: application programming interface
APN: Apple Push Notification
FDA: Food and Drug Administration
HIPAA: Health Insurance Portability and Accountability Act
HTTPS: hypertext transfer protocol secure
iOS: iPhone Operating System
mHealth: mobile health
NMES: neuromuscular electrical stimulation
PHP: hypertext preprocessor
REST: representational state transfer
SQL: structured query language

## References

[1] Griffin LY, Albohm MJ, Arendt EA, Bahr R, Beynnon BD, Demaio M, Dick RW, Engebretsen L, Garrett WE, Hannafin JA, Hewett TE, Huston LJ, Ireland ML, Johnson RJ, Lephart S, Mandelbaum BR, Mann BJ, Marks PH, Marshall SW, Myklebust G, Noyes FR, Powers C, Shields C, Shultz SJ, Silvers H, Slauterbeck J, Taylor DC, Teitz CC, Wojtys EM, Yu B (2006). Understanding and preventing noncontact anterior cruciate ligament injuries: a review of the Hunt Valley II meeting, January 2005. Am J Sports Med.

[2] Palmieri-Smith RM, Thomas AC, Wojtys EM (2008). Maximizing quadriceps strength after ACL reconstruction. Clin Sports Med.

[3] Tourville T, Jarrell K, Naud S, Slauterbeck JR, Johnson RJ, Beynnon BD (2014). Relationship between isokinetic strength and tibiofemoral joint space width changes after anterior cruciate ligament reconstruction. Am J Sports Med.

[4] Ingelsrud LH, Granan L, Terwee CB, Engebretsen L, Roos EM (2015). Proportion of patients reporting acceptable symptoms or treatment failure and their associated KOOS values at 6 to 24 months after anterior cruciate ligament reconstruction: a study from the Norwegian Knee Ligament Registry. Am J Sports Med.

[5] Thomas AC, Villwock M, Wojtys EM, Palmieri-Smith RM (2013). Lower extremity muscle strength after anterior cruciate ligament injury and reconstruction. J Athl Train.

[6] Dirks ML, Wall BT, Snijders T, Ottenbros CL, Verdijk LB, van Loon LJ (2014). Neuromuscular electrical stimulation prevents muscle disuse atrophy during leg immobilization in humans. Acta Physiol (Oxf).

[7] Gibson JN, Smith K, Rennie MJ (1988). Prevention of disuse muscle atrophy by means of electrical stimulation: maintenance of protein synthesis. Lancet.

[8] Hasegawa S, Kobayashi M, Arai R, Tamaki A, Nakamura T, Moritani T (2011). Effect of early implementation of electrical muscle stimulation to prevent muscle atrophy and weakness in patients after anterior cruciate ligament reconstruction. J Electromyogr Kinesiol.

[9] Hauger AV, Reiman MP, Bjordal JM, Sheets C, Ledbetter L, Goode AP (2018). Neuromuscular electrical stimulation is effective in strengthening the quadriceps muscle after anterior cruciate ligament surgery. Knee Surg Sports Traumatol Arthrosc.

[10] Stevens-Lapsley JE, Balter JE, Wolfe P, Eckhoff DG, Kohrt WM (2012). Early neuromuscular electrical stimulation to improve quadriceps muscle strength after total knee arthroplasty: a randomized controlled trial. Phys Ther.

[11] Bollen JC, Dean SG, Siegert RJ, Howe TE, Goodwin VA (2014). A systematic review of measures of self-reported adherence to unsupervised home-based rehabilitation exercise programmes, and their psychometric properties. BMJ Open.

[12] Hall AM, Kamper SJ, Hernon M, Hughes K, Kelly G, Lonsdale C, Hurley DA, Ostelo R (2015). Measurement tools for adherence to non-pharmacologic self-management treatment for chronic musculoskeletal conditions: a systematic review. Arch Phys Med Rehabil.

[13] Vuorenmaa M, Ylinen J, Piitulainen K, Salo P, Kautiainen H, Pesola M, Häkkinen A (2014). Efficacy of a 12-month, monitored home exercise programme compared with normal care commencing 2 months after total knee arthroplasty: a randomized controlled trial. J Rehabil Med.

[14] (2019). Pew Research Center.

[15] Quinn CC, Clough SS, Minor JM, Lender D, Okafor MC, Gruber-Baldini A (2008). WellDoc mobile diabetes management randomized controlled trial: change in clinical and behavioral outcomes and patient and physician satisfaction. Diabetes Technol Ther.

[16] Iso-Ketola P, Karinsalo T, Vanhala J (2008). HipGuard: A Wearable Measurement System for Patients Recovering From a Hip Operation. Proceedings of the 2008 Second International Conference on Pervasive Computing Technologies for Healthcare.

[17] Dunphy E, Hamilton FL, Spasić I, Button K (2017). Acceptability of a digital health intervention alongside physiotherapy to support patients following anterior cruciate ligament reconstruction. BMC Musculoskelet Disord.

[18] (2019). Piper Sandler.

[19] Bandura A (1977). Social Learning Theory.

[20] Malin B (2012). US Department of Health and Human Services.

[21] Lieber RL, Silva PD, Daniel DM (1996). Equal effectiveness of electrical and volitional strength training for quadriceps femoris muscles after anterior cruciate ligament surgery. J Orthop Res.

[22] Järvelä T, Kannus P, Latvala K, Järvinen M (2002). Simple measurements in assessing muscle performance after an ACL reconstruction. Int J Sports Med.

[23] Lautamies R, Harilainen A, Kettunen J, Sandelin J, Kujala UM (2008). Isokinetic quadriceps and hamstring muscle strength and knee function 5 years after anterior cruciate ligament reconstruction: comparison between bone-patellar tendon-bone and hamstring tendon autografts. Knee Surg Sports Traumatol Arthrosc.

[24] Beynnon BD, Johnson RJ, Naud S, Fleming BC, Abate JA, Brattbakk B, Nichols CE (2011). Accelerated versus nonaccelerated rehabilitation after anterior cruciate ligament reconstruction: a prospective, randomized, double-blind investigation evaluating knee joint laxity using roentgen stereophotogrammetric analysis. Am J Sports Med.

[25] Beynnon BD, Uh BS, Johnson RJ, Abate JA, Nichols CE, Fleming BC, Poole AR, Roos H (2005). Rehabilitation after anterior cruciate ligament reconstruction: a prospective, randomized, double-blind comparison of programs administered over 2 different time intervals. Am J Sports Med.

[26] Ardern CL, Taylor NF, Feller JA, Whitehead TS, Webster KE (2013). Psychological responses matter in returning to preinjury level of sport after anterior cruciate ligament reconstruction surgery. Am J Sports Med.

[27] Levinger P, Hallam K, Fraser D, Pile R, Ardern C, Moreira B, Talbot S (2017). A novel web-support intervention to promote recovery following Anterior Cruciate Ligament reconstruction: A pilot randomised controlled trial. Phys Ther Sport.

[28] Schippers M, Adam PC, Smolenski DJ, Wong HT, de Wit JB (2017). A meta-analysis of overall effects of weight loss interventions delivered via mobile phones and effect size differences according to delivery mode, personal contact, and intervention intensity and duration. Obes Rev.

[29] Haider R, Sudini L, Chow CK, Cheung NW (2019). Mobile phone text messaging in improving glycaemic control for patients with type 2 diabetes mellitus: A systematic review and meta-analysis. Diabetes Res Clin Pract.

[30] Uy C, Lopez J, Trinh-Shevrin C, Kwon S, Sherman S, Liang P (2017). Text messaging interventions on cancer screening rates: a systematic review. J Med Internet Res.

[31] Whittaker R, McRobbie H, Bullen C, Rodgers A, Gu Y (2016). Mobile phone-based interventions for smoking cessation. Cochrane Database Syst Rev.

[32] Armstrong AW, Watson AJ, Makredes M, Frangos JE, Kimball AB, Kvedar JC (2009). Text-message reminders to improve sunscreen use: a randomized, controlled trial using electronic monitoring. Arch Dermatol.

[33] Fischer HH, Moore SL, Ginosar D, Davidson AJ, Rice-Peterson CM, Durfee MJ, MacKenzie TD, Estacio RO, Steele AW (2012). Care by cell phone: text messaging for chronic disease management. Am J Manag Care.

[34] Horvath T, Azman H, Kennedy G, Rutherford G (2012). Mobile phone text messaging for promoting adherence to antiretroviral therapy in patients with HIV infection. Cochrane Database Syst Rev.

[35] de Tolly K, Skinner D, Nembaware V, Benjamin P (2012). Investigation into the use of short message services to expand uptake of human immunodeficiency virus testing, and whether content and dosage have impact. Telemed J E Health.

[36] Palmer M, Barnard S, Perel P, Free C (2018). Mobile phone-based interventions for improving adherence to medication prescribed for the primary prevention of cardiovascular disease in adults. Cochrane Database Syst Rev.

[37] Vodopivec-Jamsek V, de Jongh T, Gurol-Urganci I, Atun R, Car J (2012). Mobile phone messaging for preventive health care. Cochrane Database Syst Rev.

[38] Ayoade M, Baillie L (2014). A Novel Knee Rehabilitation System for the Home. Proceedings of the SIGCHI Conference on Human Factors in Computing Systems.

[39] GitHub.

[40] The University of Vermont.

